# A Training Model for Implementing Hepatitis Prevention Services in Substance Use Disorder Clinics: A Qualitative Evaluation

**DOI:** 10.1007/s11606-015-3317-3

**Published:** 2015-04-23

**Authors:** Hildi J. Hagedorn, Nancy Rettmann, Eric Dieperink, Astrid Knott, Bruce E. Landon

**Affiliations:** Substance Use Disorders Quality Enhancement Research Initiative, Minneapolis VA Health Care System, One Veterans Drive (152), Minneapolis, MN 55417 USA; Department of Psychiatry, School of Medicine, University of Minnesota, Minneapolis, MN USA; Department of Psychiatry, Minneapolis VA Health Care System, Minneapolis, MN USA; Department of Health Care Policy, Harvard Medical School, Boston, MA USA; Division of General Medicine and Primary Care, Beth Israel Deaconess Medical Center, Boston, MA USA

**Keywords:** Implementation, Hepatitis, Prevention, Substance Use Disorders

*Editors’ note: In this installment of Implementation Science Workshop, Dr. Hagedorn and colleagues describe implementation and evaluation of a liver health training program at the Minneapolis VA. In an accompanying commentary, Bruce Landon, MD, MBA, MSc, of Harvard Medical School highlights the distinction between evaluating the process and effectiveness of an intervention, important components of implementation science.*

**CASE**

## Introduction

Drug- and alcohol-dependent individuals are at a much higher risk for hepatitis infections than the general population and are at higher risk of poor outcomes following infection.^1^–^13^ Because of these risk factors, substance use disorder treatment clinics represent an ideal venue for screening, education, prevention, and treatment referral services for patients with hepatitis infections.

The Liver Health Initiative (LHI) was a training program jointly sponsored by the Veterans Health Administration’s (VA) Substance Use Disorders Quality Enhancement Research Initiative (SUD QUERI) and the Minneapolis VA Hepatitis C Resource Center (HCRC), with the goal of implementing these services in VA SUD clinics. The program was based on a successful liver health program established in the Minneapolis VA Health Care System’s Addictive Disorders Service^14^ and a training model previously used by the HCRC to promote evidence-based evaluation and treatment of patients with hepatitis C.^15^ The program design was informed by empirically based literature on the education of medical providers and the dissemination of health care innovations.^16^–^22^

The purpose of this paper is to describe the LHI training model, which shows promise for promoting implementation of hepatitis services in SUD clinics and may also be generalizable to promoting implementation of other evidence-based health care practices. The evaluation of this initial cohort of LHI trainees builds on the previous evaluation by assessing the generalizability of the effectiveness of this training program to a new topic area and target audience, comparing the intervention teams to wait-list teams receiving informational materials only, and collecting qualitative data regarding barriers, facilitators and strategies utilized to provide information about team or organizational characteristics that affect change. We hypothesized that 1) the training program would be effective in promoting implementation of recommended practices, 2) teams attending the training program would demonstrate greater success with implementation compared to wait-listed teams, and 3) the degree of implementation success would vary among teams attending the training, with qualitative data providing exploratory information on potential team and organizational characteristics that promote successful implementation.

## Settings and Participants

### Study Design

Teams from VA SUD clinics volunteered to participate in the LHI training program in response to advertisements on the VA national addictions email group and conference calls. Teams were randomly assigned either to participate in the training program or to be placed on a waiting list and provided with printed educational materials. A representative from each training team and wait-listed team was interviewed at 1, 3, and 6 months post-intervention on self-reported implementation success as well as implementation strategies, barriers, and facilitators. This study was reviewed and approved by the Minneapolis VA Health Care System’s institutional review board.

### Participants

Applicants were required to apply as a team including a representative of SUD or mental health service leadership, a front-line SUD provider with an interest in providing liver health services, and a hepatitis clinician. Applicants from 25 VA medical centers across the United States applied to participate. Of the 17 facilities that applied with a full team, 11 were randomly selected to participate in the training program. The remaining six teams were informed that they were on a waiting list for the next training program and were sent a training binder containing all materials distributed at the training. All wait-listed teams were asked to complete study evaluations, and five of six agreed. All training and wait-listed teams were located within large medical centers in urban areas. Online Appendix 1 presents the clinic roles of team members, number of patients served and staffing levels of the SUD clinics, and location and complexity of the medical facilities in which the SUD clinics were located. All VHA facilities receive a complexity rating based on the clinical services offered, characteristics of the patient population served, and educational and research mission, with 1A representing the most complex medical facilities and 3 representing the least complex. (See Online Appendix 2 for details on complexity rating methodology.)

## Program Description

Both training and wait-list teams were sent a Needs Assessment Questionnaire 6 weeks prior to the 1.5-day training (see Online Appendix 3). The questionnaire was designed to evaluate current clinic practices regarding the five implementation goals listed in Table 1. As shown in Table 1, the teams had one or more goals in place at baseline. However, most teams had several, and all had at least one implementation goal that they could work on. Needs Assessment information was utilized during the Action Planning phase of the 1.5-day training to assist the training teams in developing concrete implementation goals that would address gaps between baseline and recommended practice. Teams were strongly encouraged, but not required, to address all unmet goals in their Action Plan.

**Table 1:** Recommended Practices and Definitions

Practice recommendation	Definition	Intervention clinics with goal met at baseline (Clinic ID)	Wait-list clinics with goal met at baseline (Clinic ID)
1. Routine screening for HCV, HBV, HAV, and HBV and HAV immunity	Every patient presenting for intake is screened as part of routine intake laboratory workup.	None	Clinics 8, 15
2. Routine feedback of screening results	A designated provider is responsible for reviewing and communicating laboratory results at a designated appointment.	Clinics 1, 2, 4, 5, 6, 7, 9, 11, 12, 14	Clinics 8, 10, 13, 15, 16
3. Expedited follow-up in specialty clinic (e.g., gastroenterology, liver or hepatitis clinic)	Patients testing positive are referred directly and immediately to a specialty clinic at the time the results are communicated.	Clinics 4, 6, 11	Clinics 8, 15
4. HAV/HBV vaccinations available and routinely offered in SUD clinic	Patients are offered vaccination, as appropriate based on screening results, at the time results are communicated.	None	None
5. Routine availability of comprehensive hepatitis education	All new patients are scheduled into a stand-alone hepatitis education class.	Clinics 2, 5, 9, 12, 14	Clinics 8, 16

The first day of the training consisted of didactic presentations and discussion periods covering the risks for liver disease among patients with SUDs, the goals of the LHI, and implementation strategies. The second day was devoted to assisting teams in developing site-specific action plans. Each team was presented with an Action Plan form with implementation goals and Needs Assessment information regarding the baseline practice that they had recorded. Teams were encouraged to list an improvement goal and specific actions that they could commit to within the next month for each row in which the baseline practice did not match the implementation goal.

As part of the training intervention, one representative from each training team was contacted by phone 1, 3, and 6 months after the training. The representative was required to be an SUD clinic staff member with close knowledge of daily processes within the clinic, and was generally the SUD clinic coordinator or an SUD nurse. The purpose of these coaching calls was to assess progress on Improvement Goals and Action Steps recorded in the Action Plan and to coach the team in implementing their plans.

## Program Evaluation

### Methods

Semi-structured interview follow-up assessments were completed at 1, 3, and 6 months post-training for both the training and comparison teams. At each assessment, teams were evaluated on whether they had achieved each of the implementation goals that had not already been fully in place at baseline. The designated team representative reported on current clinic practices. Table 1 describes the practices that were required to be in place to receive “credit” for the implementation of each goal.

Implementation success was determined by the number of implementation goals achieved by the 6-month follow-up assessment. Teams only received “credit” for achieving implementation goals that had not already been in place at baseline. Because teams had different numbers of services in place at baseline, consideration was given to counting all services in place. However, because the focus of the training was on implementing new practices, it was ultimately decided that this would better reflect the impact of the program. Only two teams had more than two services in place at baseline, so most teams had the opportunity to demonstrate a high degree of change.

Implementation strategies used (e.g., actions taken by team members to implement goals) were assessed based on responses to open-ended interview questions. Respondents were also asked to report on perceived local barriers and facilitators with regard to the changes they were attempting to implement (e.g., what helped or hindered their implementation efforts). Researchers analyzed responses using a standard qualitative analysis technique that consisted of creating a code list with categories of major themes and coding text into the major categories.^23^ Coders (HH & NR), who were blind to site, condition, implementation success, and time point (1-, 3-, or 6-month follow-up), collaboratively created a list of codes within the broad categories of strategies, barriers, and facilitators, as well as additional codes as relevant from the interviews. The two original coders plus a third coder then held coding meetings in which they reviewed their independently created codes and resolved any inconsistencies collaboratively. Once all documents were coded and inconsistencies were resolved, coders were un-blinded to team condition and implementation success for data analysis. Clinics were grouped according to *high*, *moderate*, *low*, or *no* change, and strategies, barriers, and facilitators reported by the teams were examined to assess for differences in type and frequency of codes reported.

### Results

The results indicated that teams fell into four distinct groups: 1) those that had few services in place at baseline and implemented three to five recommendations (*high)*, 2) those that had some services in place at baseline and implemented two additional services (*moderate)*, 3) those that added one new service (*low)*, and 4) those that did not implement any practice change (*none)*. Results showed that the training was effective in promoting implementation of recommended practices, with nine of 11 intervention teams (82 %) achieving *high* to *moderate* implementation success. The results also confirmed that the training was more effective than receiving printed materials, with all wait-listed teams falling into the *low* or *no* change categories. Categorizing by total services in place rather than new services implemented would have resulted in one intervention team and one wait-list team being rated more highly. Finally, there is clear variability in implementation success among teams attending the training, ranging from 0 to 5 new services implemented. The remainder of the results explore reported implementation strategies, barriers and facilitators, and their potential relationship to implementation success.

Table 2 displays a summary of the strategies, barriers, and facilitators reported by each clinic. On average, *high* and *moderate* change teams reported 5.6 strategies, while *low* change teams reported 3 strategies and *no* change teams reported 2 strategies. The most frequently cited strategies used to implement change included the local team established for the training program holding team meetings, meeting with additional SUD clinic staff, meeting with hepatitis clinic staff, or meeting with representatives from administration. With the exception of holding training program team meetings, these strategies were employed by most teams regardless of degree of change. Additional strategies were utilized predominantly by *high* and *moderate* change teams.

**Table 2:** Strategies, Barriers and Facilitators Reported by Clinics

Change category	High				Moderate					Low			None			
Clinic	3	7	1	12	2	4	6	9	14	5	10	13	8	11	15	16
Condition*	I	I	I	I	I	I	I	I	I	I	W	W	W	I	W	W
Strategies																
Local training team meetings	X	X	X	X	X	X	X	X	X	X						
Training team meets with other SUD staff	X		X		X	X			X	X	X	X	X		X	
Training team meets with other hepatitis clinic staff	X		X	X		X		X	X		X	X	X		X	
Training team meets with facility administration	X		X	X	X			X	X		X		X		X	
Provide in-service for SUD clinic staff	X	X	X	X	X	X		X	X						X	
Provide in-service for hepatitis clinic staff			X	X						X						
Contact with other preceptorship participants	X				X			X								
Collaborate with additional services (e.g., lab, pharmacy)	X								X							
Create standardized templates for notes, consults or orders	X	X		X												
Collect baseline data to guide goal development/track progress				X	X			X								
Utilize materials provided by project (e.g., protocols, educational video)				X			X		X			X	X			
Barriers																
Lack of time/competing priorities	X		X	X	X	X		X	X	X	X	X	X	X	X	
Not enough staff			X	X	X				X			X	X	X	X	X
Poor collaboration with the hepatitis clinic				X		X			X	X			X	X		X
Patient issues that interfere with appropriate follow-up (e.g., homelessness, no-shows)	X	X		X	X		X						X			X
SUD clinic in physically separate location from hepatitis clinic				X	X				X							X
Technology issues that prevented use of training materials				X		X							X			
Incompatible procedures in other clinics				X				X	X			X				X
Lack of support from facility administration				X	X	X							X			
Lack of knowledge about hepatitis among SUD staff						X								X		X
Lack of support from SUD clinic staff						X			X				X			
Budget constraints						X			X					X		
Facilitators																
Strong collaboration with hepatitis clinic		X			X		X	X			X	X			X	
Interested and knowledgeable staff	X	X					X	X		X					X	
Felt supported by facility and/or VHA national administration	X			X												
Received help from other services (e.g., lab, pharmacy)	X			X	X			X								
Gained additional staff to assist with implementation			X			X	X	X								
Coaching calls			X													

* I = Intervention, W = Wait-List

On average, *high* and *moderate* change teams reported 3.7 barriers, while *low* change teams reported 2 barriers, and *no* change teams reported 5 barriers. The most frequently cited barriers to implementing change included lack of time and competing priorities, not enough staff, poor collaboration with the hepatitis treatment clinicians, and patient issues that interfered with appropriate follow-up (e.g., no-shows, homelessness).

On average, *high* and *moderate* change teams reported 2.1 facilitators, while *low* change teams reported 1 facilitator and *no* change teams reported 0.5 facilitator. The most frequently cited facilitating conditions for implementing change included a strong collaboration with the hepatitis clinic and having interested and knowledgeable staff in the SUD clinic beyond team members. These were reported by many teams across all change categories. Additional facilitators were cited only by *high* and *moderate* change teams.

## Challenges & Future Plans

The Minneapolis VA HCRC training program was originally developed for hepatitis treatment clinicians to promote evidence-based evaluation and treatment of patients with hepatitis C. A previous evaluation demonstrated the effectiveness of the program in improving clinic processes and promoting organizational change.^22^ For the LHI, the original structure of the training program was retained, while the specific content was revised to address a different population and a different goal. Results indicate that the effectiveness of the training model is generalizable to this new population and goal, and that the training program is superior to receiving printed training materials. The results also reveal substantial variability in implementation success among the training teams.

As the number of changes implemented increased, the number of strategies and facilitators reported also increased. While an inverse relationship with barriers might be expected (e.g., more barriers reported, fewer changes implemented), there was no consistent relationship.

Looking at facilitators, it is notable that the teams that were more successful at implementing practice change were more likely to endorse support from the broader system beyond the SUD clinic (e.g., receiving additional staff to assist with implementation, receiving help from other services when requested, and feeling supported by the hospital administration). This suggests that the effectiveness of the training program could be further improved if efforts were made to solicit support from medical center leadership.

Training teams employed a greater number of strategies, and the types of strategies were qualitatively different. All but one training team (also the only training team in the *no* change category) held formal LHI team meetings, while none of the comparison teams held such meetings, despite forming a team for submission of their LHI application. In addition, training teams were more likely to use active implementation strategies—strategies that required effort beyond convening meetings, such as developing and providing in-service training, developing standardized templates for notes or orders, or establishing a data collection process to monitor progress on goals. These findings suggest that a functional team and active change strategies are vital ingredients for making changes in clinic practice. Only three teams reported having any contact with training teams from other facilities despite having access to contact information for other teams. The impact of the training program could have been improved by facilitating greater interaction among teams following the training to share successful strategies.

These findings provide preliminary support for the value of specific components of the training program aimed at developing a functional team, educating teams in specific active change strategies, and walking them through several steps of the implementation process.

Limitations in the methods of data collection affect the generalizability of these results. Implementation success was reported by one representative from each team, who clearly knew whether their team had been selected to attend the training or wait-listed. This awareness may have created demand characteristics encouraging the training team representatives to over-report their success. Credibility of the reports could have been improved by gathering an independent report from another team member or from an SUD staff member who was not part of the training team. Also, while implementation of a new service had to meet the minimum standards listed in Table 2 to receive “credit”, it is possible that some teams may have implemented higher-quality services than others. Perhaps prioritizing implementation of one or two high-quality services would have greater impact on patient outcomes than focusing on the entire package of services. Since patient-level outcomes were not collected, this remains unknown. In addition, while the training teams were randomly selected from the applicants, wait-listed teams self-selected to complete the project evaluations. Although five of six agreed to complete the evaluations, it is not clear how those that agreed may have differed from the program that declined. Finally, participating clinics were embedded in large VA medical facilities. Generalizability to community SUD clinics would require further evaluation.

In the future, it will be necessary to assess the impact of practice change prompted by this training model on patient-level outcomes such as screening and vaccination rates. To date, all evaluation of the model has focused on practice change, which is assumed to translate into improved patient-level outcomes. It will also be necessary to evaluate the effectiveness of this training model in areas distinct from hepatitis care in order to establish the model as a generalizable method to improve the quality of medical care.

**TEACHING COMMENTARY**

By Bruce E. Landon, MD, MBA, MSc

This paper describes and evaluates a quality improvement program that takes the form of a discrete educational intervention (the Liver Health Initiative) aimed at improving screening and treatment for hepatitis for VA patients enrolled in substance use disorders (SUD) clinics. To understand its effectiveness, the authors evaluated the first cohort of SUD centers that completed the training module.

In this commentary, I outline the importance of including both “process” and “outcomes” components in evaluations of QI interventions, with a particular focus on understanding the importance of knowing both *whether* the intervention was successful overall and *how* the innovation was implemented at various clinics.^24^ In addition, I comment on other design aspects of the intervention that contribute to the usefulness of the evaluation, including the use of mixed methods and the reliability and validity of relying on informant interviews to understand whether an intervention was effective.

## Evaluation Design

This largely qualitative evaluation consisted primarily of telephone interviews conducted with a clinic representative at 1, 3, and 6 months asking about post-implementation success as well as facilitators and barriers. Although used in the evaluation, the calls also were part of the intervention in that they were used as opportunities to coach the teams in implementation of their plans. Teams were categorized according to the extent to which they added services, with 9 of 11 intervention teams rated as achieving high to moderate implementation success.

## Mixed Methods Research in Implementation Science

In implementation science, it is important both to understand the effectiveness of a particular intervention (e.g., in this case, *whether* the educational program improved treatment) as well as to understand *how* the outcome was achieved by understanding the specific changes made at clinics in order to affect care, as well as the extent to which clinics adhered to the recommendations of the QI intervention.

In addition, feedback on the implementation of a model can also help inform the evolution of the model and improve it over time. Such innovations are rarely “fixed,” in that there are always ways to improve or evolve the model, or adapt it to local circumstances through experiential learning.^25^ In fact, the Institute for Health Care Improvements’ collaborative model for achieving breakthrough improvement teaches methods of quality improvement including plan/do/study/act cycles, but leaves it to individual clinics to identify strategies and tactics that might work in their own environments.^26^,^27^ Thus, evaluations can help determine what specific aspects of the program were implemented in order to explain *how* an overall result was achieved.

## Understanding *Whether* an Intervention Works

The first important step to understanding the impact of any QI intervention is to evaluate the impact of the intervention on patient care and outcomes. Even if the educational intervention was reported as effective by participants, its ultimate success as a quality improvement intervention will be defined by the extent to which patients at the enrolled clinics received the recommended screening tests and treatments.

In this case, the main results of the evaluation are based on reports from a single informant from each clinic who reported whether the clinic had implemented each of the recommended practices within 6 months, but the evaluation failed to address whether the intervention effectively improved care. This approach also raises questions about the reliability and validity of such reports. Reliability is defined as the extent to which a particular characteristic (in this case, of a clinic) is measured consistently, whether, for example, as reported by different respondents (inter-rater reliability) or by the same respondent over time (test-retest reliability). Reports on organizational practices from a single informant tend not to be highly reliable unless the respondent is reporting on basic structural characteristics. For instance, in an earlier study, we found that a single respondent could accurately report the number of physicians in a practice, but in order to obtain reliable measures of clinic operational characteristics, more responses were required than there were members of a typical clinic.^28^ Thus, relying on a single informant often can be problematic, particularly for aspects of clinical management that might not be applied uniformly across a clinic.

Validity refers to the extent to which responses reflect what is actually happening in the clinic. In this case, there also were reasons to question the validity of the responses. Respondents participating in the QI program likely would have been biased to provide responses that conformed to the tenets of the educational program, whereas those who were in the control group would have been less likely to endorse these activities simply as a result of being in the program.^29^,^30^ Thus, this level of evidence might be suggestive of the success of the program, but should only be considered as hypothesis-generating, and more objective evidence would be required before concluding that the program was effective.

The investigators might consider alternative approaches in order to understand more definitively *whether* the program worked. A common approach that would allow for such inferences would be to collect actual clinical data, and there are several potential study designs that would allow for this type of effectiveness evaluation. In many QI interventions, clinics are instructed to create registries and to record the results of tests and interventions. In this case, the intervention is aimed at new enrollees to the clinic (which ranges from approximately 5 to 50 new patients per month across clinics). Thus, it would not be particularly burdensome for clinics to create a registry to track the actual outcomes for each of the newly enrolled patients. A limitation to this approach, however, is that control clinics would not be collecting these data, and if they were asked to as part of the study, the mere collection of data could influence their results. In addition, if some patients were excluded from the registries, this could lead to inaccurate or biased results.

A better design would be to collect these data via other means. With sufficient funding, chart reviews could be conducted at both intervention and control sites. Alternatively, it also is possible that these data could be collected passively—in this case, through the VA health care system’s unified electronic medical record if one could identify the patients being seen in the clinics.^31^ In this case, the ultimate goal of this program is to perform laboratory tests and vaccinate patients as needed and to make sure that those who screen positive are reliably referred into care, all of which can be ascertained through the EMR. Either of these approaches would provide the strongest evidence of the success of the program. In addition, researchers also could passively access other potential SUD treatment sites to expand the candidate pool of control centers.

Text Box 1. Teaching Points for Quality Improvement Evaluations

**Table:** 

• Quality improvement evaluations should include both “process” and “outcomes” components in order to understand both *whether* the intervention worked and *how* it worked.
• Mixed methods approaches are well suited to addressing these questions.
• Evaluation designs should carefully consider both potential biases related to the design and the reliability and validity of the data used in the evaluation.

## Understanding the *How* of an Intervention

As opposed to the controlled settings of clinical trials, most QI interventions are implemented in the “messy” world of actual clinical practice. As noted by others, QI interventions seek to change behavior rather than create new scientific knowledge, and learning in QI is driven by experiential learning.^32^ Moreover, improvement efforts are context-dependent, and both the interventions and the outcomes can be modified over time based on feedback.^33^ Thus, qualitative interview techniques such as those used in this study are well suited to learning about the nuances of an intervention and the potential barriers and facilitators to implementation. Thus, once it is known whether an intervention is effective, qualitative techniques are useful for understanding the *how* of the intervention—the mechanism, the specific populations, and the settings where it is most effective.

Such techniques also can inform efforts to redesign or improve interventions. Evaluations can be used to assess the extent to which clinics adhered to the intervention, and can potentially identify aspects of the program that were more or less likely to be adopted by participating clinics. For instance, in the current study, the interviews assessed whether the clinics addressed each of the recommended practices at 6 months. Thus, such interviews can provide data on how well the intervention was being implemented and aspects of the intervention that seemed to work better or worse. Such information would be useful for potentially redesigning the intervention.

In addition, qualitative interviews can be particularly effective for identifying barriers to implementation as well as aspects of clinics that might have facilitated the intervention. Such knowledge can be useful in propagating interventions to other clinics, whether by informing the selection process by focusing on clinics that lacked specific barriers or by helping to inform changes in the program that might overcome barriers or take advantage of facilitating factors. Issues such as the lack of time, staffing, or attention such as seen in this study might lead designers of interventions to require specific commitments of resources from senior management, whereas challenges such as poor collaboration with the hepatitis clinic might require active participation of health system leaders.

Qualitative interviews also can provide insight into how particular characteristics might have influenced the success of the implementation. In this study, participating clinics ranged in size (including both the number of staff and number of patients treated) as well as organizational structure, and some of these might have been more conducive than others to accomplishing the goals of the QI project. In particular, receiving support, including both resources and buy-in from the greater health system, was seen as important to the success of individual clinics.

Thus, understanding both *whether* an intervention was effective and *how* it worked are crucially important in implementation science. Both aspects are required in order to allow readers to understand how a particular intervention might work in their own context and to help guide the investment of their scarce resources.

## Electronic supplementary material

Below is the link to the electronic supplementary material.

ESM 1 (DOCX 22 kb)

ESM 2 (DOCX 22 kb)

ESM 3 (DOCX 48 kb)
