# Supplementary material for: A Training Model for Implementing Hepatitis Prevention Services in Substance Use Disorder Clinics: A Qualitative Evaluation
Source: J Gen Intern Med. 2015 Apr 23;30(8):1215–21. doi: 10.1007/s11606-015-3317-3 (PMC4510217; doi:10.1007/s11606-015-3317-3)
Supplement: Supplementary file 1 — (DOCX 22 kb) [file 11606_2015_3317_MOESM1_ESM.docx]

Appendix 1: Clinic and Team Characteristics

| **Clinic ID** | **Condition** | **Team Members: Clinic Role (Degree*)** | **Location:**  **Census Region** | **Facility Complexity†** | **Staffing (FTE)‡** | **New Patient Intakes Past Month** |
| --- | --- | --- | --- | --- | --- | --- |
| 1 | Intervention | SUD Medical Director (MD); SUD Nurse (NP); Hepatitis C Clinic Coordinator (RN) | South | 1B | 7 | 40 |
| 2 | Intervention | SUD Medical Director (MD); SUD Nurse (RN); Hepatitis C Clinic Coordinator (CNS) | Northeast | 1A | 4.4 | 3 |
| 3 | Intervention | Mental Health Service Line Chief Nurse (RN); SUD Nurse (NP); Hepatitis C Nurse (NP) | Midwest | 1B | 6 | 45 |
| 4 | Intervention | SUD Medical Director (MD); SUD Nurse (RN); Hepatitis C Nurse (NP) | South | 1A | 6 | 20 |
| 5 | Intervention | SUD Clinic Coordinator (CNS); SUD Nurse (CNS); Chief of Hepatology (MD) | South | 1B | 18 | 30 |
| 6 | Intervention | SUD Clinic Coordinator (MSW); SUD Nurse (RN); Hepatitis C Physician (MD) | South | 1C | 6 | Not reported |
| 7 | Intervention | SUD Medical Director (MD); SUD Provider (PA); Hepatitis C Clinic Coordinator (PA) | Midwest | 1C | 14 | 20 |
| 8 | Wait-List | SUD Clinic Coordinator (MSW); SUD Addiction Therapist (not reported); Hepatitis C Nurse (NP) | South | 2 | 15 | 40 |
| 9 | Intervention | SUD Clinic Coordinator (CNS); SUD Nurse (NP); Hepatitis C Clinic Coordinator (CNS) | Midwest | 1A | 7.7 | 6 |
| 10 | Wait-List | SUD Clinic Coordinator (not reported); SUD Nurse (NP); Hepatitis C Provider (not reported) | Midwest | 1A | 1 | Not reported |
| 11 | Intervention | SUD Medical Director (MD); SUD Provider (not reported); Hepatitis C Nurse (NP) | West | 1A | 4.15 | Not reported |
| 12 | Intervention | SUD Medical Director (MD); SUD Nurse (BNS); Hepatitis C Clinic Coordinator (RN) | West | 1A | 7 | 3 |
| 13 | Wait-List | SUD Clinic Coordinator (NP); SUD Nurse (RN); Hepatitis C Provider (not reported) | South | 1A | 8.7 | 40 |
| 14 | Intervention | SUD Clinic Coordinator (CNS); SUD Nurse (NP); Director of Gastroenterology (MD) | South | 2 | 9.25 | 16 |
| 15 | Wait-List | SUD Medical Director (MD); SUD Case Manager (BSN); Hepatitis C Provider (PA) | South | 1A | 20 | 45 |
| 16 | Wait-List | SUD Medical Director (MD); SUD Nurse (RN); Hepatitis C Provider (not reported) | Midwest | 1A | 11 | 57 |

*MD=Medical Doctor; NP=Nurse Practitioner; RN=Registered Nurse; CNS=Clinical Nurse Specialist; MSW=Masters of Social Work; PA=Physicians’ Assistant; BNS=Bachelors of Nursing Science

†1=High Complexity (A representing highest level in this category followed by B and C); 2=Medium Complexity; 3=Low Complexity

‡FTE=Full Time Equivalent
