# Supplementary material for: A Training Model for Implementing Hepatitis Prevention Services in Substance Use Disorder Clinics: A Qualitative Evaluation
Source: J Gen Intern Med. 2015 Apr 23;30(8):1215–21. doi: 10.1007/s11606-015-3317-3 (PMC4510217; doi:10.1007/s11606-015-3317-3)
Supplement: Supplementary file 2 — (DOCX 22 kb) [file 11606_2015_3317_MOESM2_ESM.docx]

Appendix 2: Facility Complexity Level Model Category Descriptions and Variable Definitions

The Facility Complexity Model relies on seven variables that are compiled at the facility level as described below:

Variable 1: VERA Pro-Rated Person (PRP): The Allocation Resource Center calculated PRP assigns patients to facilities based on the amount and complexity of care received at each facility. This measures workload and also accounts for patient sharing and workload complexity of shared patients.

Variable 2: ICU/VHA Operative Complexity Level: The facility ICU/Operative Complexity Level measures the general level of complexity of both the intensive care unit and the operative surgical program at each facility. The ICU level and Operative Complexity level were combined such that a facility with the highest score possible is a facility with Level 1 ICU with complex surgery and lowest score possible is a facility with neither program.

Variable 3: Patient Risk: Patient risk is measured by diagnostic cost group (DCG) score at the facility level. This is a Medicare relative risk score calculated from all VA patient diagnosis from all sources. In places where the population is sicker, the management of that health care system will be more difficult because of the complexity of illnesses associated with sicker patients.

Variable 4: Total Resident Slots: This is a count of resident slots as a measure of education mission. The more resident slots a facility has, the bigger the teaching program is at the facility. Maintaining a large teaching program adds to the complexity of managing the facility.

Variable 5: Herfindahl-Hirshman Index: This Index accounts for multiple residency programs at a single facility. This metric rewards facilities with multiple large residency programs because the diversity in the number of residency programs increases the complexity of managing a facility’s educational mission (e.g., a facility with 100 residents in 2 programs is more difficult to manage than a facility with 100 residents in 1 program).

Variable 6: VERA Research Dollars: This is the total amount of VERA research dollars allocated to a facility. VERA research dollars is used as a proxy measurement for how large the research mission is at that facility. Large research programs increase the complexity of managing a facility because of the compliance requirements, additional staff, space, and monitoring required for managing these programs.

Variable 7: Complex Clinical Programs: Complex clinical programs are associated with specialized clinical services that require increased administrative responsibilities, reporting, accreditation, congressional oversight, and other unique requirements making management of a facility with such in-house programs more complex than a facility without the programs. These programs also are usually associated with specialized equipment, staff, or space needs in order to operate the programs. Complex clinical programs include: Spinal Cord Injury, Blind Rehabilitation, Cardiac Surgery, Invasive Catheter Laboratory, Neurosurgery, Transplant, Radiation Oncology, Interventional Radiology, Polytrauma, Inpatient/Acute Mental Health and Post-Traumatic Stress Disorder, and Mental Health Intensive Case Management.

Summary: Data components of each of the seven variables are collected for all facilities and then indexed (facility score – VHA mean/VHA standard deviation) to get a score for each facility for each variable. The scores are summed to get the facility overall scores. These scores are charted, and based on reasonable breaks in the distribution, complexity levels are assigned.

(Information summarized from the Department of Veterans Affairs, Veterans Health Administration, Executive Decision Memo: 2011 Facility Complexity Level Model)
