# Supplementary material for: A Training Model for Implementing Hepatitis Prevention Services in Substance Use Disorder Clinics: A Qualitative Evaluation
Source: J Gen Intern Med. 2015 Apr 23;30(8):1215–21. doi: 10.1007/s11606-015-3317-3 (PMC4510217; doi:10.1007/s11606-015-3317-3)
Supplement: Supplementary file 3 — (DOCX 48 kb) [file 11606_2015_3317_MOESM3_ESM.docx]

Appendix 3: Needs Assessment Questionnaire


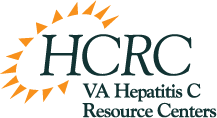


Liver Health Training Program

Minneapolis VAMC

Needs Assessment Questionnaire

Name: _________________________ ________________

Last First

Site: ________________________

Instructions:

1. Please complete this form and fax it to: Nancy Rettmann at 612-467-5312. *Your participation in the Liver Health Training Program will be confirmed upon receipt of this needs assessment questionnaire.*

2. Please review and respond to each of the following questions.
*There is no right or wrong answer. This form is meant to help you consider the services you provide in your clinic related to hepatitis infections and will help you develop an action plan to implement the most important changes you wish to see in your clinic.*

**Clinic Demographics**

1. Staffing: Complete the following table describing the number of FTE that currently work in your program.

**Title Number of FTE**

Psychiatrists _________

Psychologists _________

Social Workers _________

Addiction Therapists _________

Nurse Practitioners (NP) _________

Clinical Nurse Specialists (CNS) _________

Registered Nurses (RN; other than CNS/NP) _________

Licensed Practical Nurses (LPN) _________

Clerical Staff _________

Other: _________

1. Do you feel that you have adequate staff to manage your current work load?

1. Yes

2. No

Comments:

____________________________________________________________

1. What types of treatment are available to your clients? (circle all that apply)

1. Inpatient

2. Residential treatment

3. Intensive outpatient treatment

4. Case management

5. Aftercare groups

6. Opioid replacement treatment

7. Community support groups

8. Psychiatric consultation/management

9. Detoxification

10. Other: (describe)__________________________________

1. List the number of patients seen in your clinic in an average month:
2. Number of new intakes in a month: _________________________
3. Number of patients in intensive treatment (e.g., 2 or more groups or individual appointments per week): _____________________
4. Number of patients in aftercare or case management (e.g., one appointment per week or less): ________________________________________
5. Does your clinic have a standard intake procedure for new patients?
6. Yes
7. No
8. What procedures are completed during the intake process? (Circle all that apply)

1. History and diagnostic interview

2. Physical exam

3. Urine screening

4. Lab work

5. Other: (describe)______________________________________

**Screening**

1. Does your clinic test patients for:

Hepatitis C?

- - 1. Routinely
    2. If requested by patient
    3. At clinician’s discretion
    4. No
    5. Other_________________________

Hepatitis B?

1. Routinely
2. If requested by patient
3. At clinician’s discretion
4. No
5. Other__________________________

Immunity to hepatitis B?

1. Routinely
2. If requested by patient
3. At clinician’s discretion
4. No
5. Other__________________________

Immunity to hepatitis A?

1. Routinely
2. If requested by patient
3. At clinician’s discretion
4. No
5. Other__________________________

**(If no testing done in clinic, skip to question 10)**

**Feedback and Referral**

1. How are lab test results communicated to patients who are tested? (Circle all that apply.)
2. Patients receive test results at follow-up appointment
3. Patients receive test results by mail
4. Patients receive a phone call to communicate test results
5. No specific procedures in place
6. Other____________________________
7. What is the follow-up plan for patients who test positive for hepatitis C or
   hepatitis B?
8. Test results are communicated to patient and patient is responsible for seeking follow-up.
9. Patient is referred to primary care for follow-up
10. Patient is referred to a specialty clinic (e.g., gastroenterology, hepatology) for follow-up
11. Patient must be abstinent from drugs and alcohol for a specified period of time before a treatment referral is made. How long? _____________________________
12. Other_______________________________

**Vaccinations**

1. Are vaccinations for hepatitis A and B offered in your clinic?
2. Hepatitis B only
3. Hepatitis A only
4. Both
5. Neither

**Education**

1. Does your clinic provide education for clients regarding hepatitis transmission/ prevention?
2. Yes, to all clients
3. Yes, to clients in certain programs

(Specify:______________________________________________________________________________________________________________________________)

1. No *(skip to question 16)*
2. If yes, in what format? (Circle all that apply)
3. Group session
4. Individual session
5. Reading materials
6. Video
7. Other ____________
8. Please, check topic areas that are covered:

| □ | How hepatitis is contracted |
| --- | --- |
| □ | The importance of testing for hepatitis C |
| □ | How hepatitis C differs from hepatitis A and hepatitis B |
| □ | Importance of receiving hepatitis A and B vaccinations if you have hepatitis C |
| □ | Possible consequences of untreated hepatitis C |
| □ | How to avoid contracting hepatitis C infection if you are hepatitis C negative |
| □ | How to avoid transmitting hepatitis C if you are hepatitis C positive |
| □ | How to maintain health if you are hepatitis C or hepatitis B positive |
| □ | The effects of alcohol use in relation to hepatitis C |
| □ | Rates of hepatitis C among drug users |
| □ | Treatment for hepatitis C |

**14) If you currently conduct a group hepatitis C education class in your substance abuse treatment program, do you use any of these VA National Hepatitis C Program/Hepatitis C Resource Centers (HCRC) materials for educating patients?**

(check **ALL** that apply)

- Hepatitis C Education Class slide set (PowerPoint)
- Hepatitis C Education Slideshow with Audio
- Hepatitis and Your Liver Video
- Brochures
- We use our own materials
- None

**15) If you currently use the Hepatitis C Education Class Slide Set (PowerPoint), would you derive any benefit by having an HCRC staff member help you modify the slides to better meet the specific needs of your patients?**

- 1. Yes
  2. No *(skip* *to question 16)*

**15B) If “Yes,” please provide information so we can contact you about modifying the slides:**

Name:

VA Facility/Location:

**16) If “No,” what are the barriers that make it difficult to have such a class?** (Check **ALL** that apply)

- No interested provider
- No provider with adequate expertise / training in HCV
- No provider with adequate expertise / training in classes / groups
- Lack of provider time (too busy with other duties)
- Poor patient attendance in past
- Fear of violating patient confidentiality
- Not enough patients at a given time to justify a class
- No materials, curriculum, or slideset
- No available space large enough

**17)** **If “No,” which type of provider would be most likely to start a hepatitis C education class at your facility?** (Check **ONLY ONE**)

- Nurse / Nurse Practitioner
- Substance Abuse Counselor
- Hepatologist / Liver Specialist
- Social Worker
- Psychologist
- Psychiatrist

18) If “No”, do you believe that you or someone in your facility would benefit from communicating with an HCRC staff member who could help you facilitate starting an HCV patient education class?

1. Yes
2. No

19) If “Yes,” please provide information so we can contact this person about starting such a group:

Name:

VA Facility/Location:

**If “Yes,” what do you need to establish a hepatitis C education class at your facility?**

**Collaboration**

20) What department in your facility is responsible for treating hepatitis C?

1. Gastroenterology
2. Hepatology
3. Primary Care
4. Other: (specify)________________________________

21) Do you receive referrals of hepatitis C patients with substance use issues from the department identified in Question 16?

1. Yes: Approximately how many per month? ________________________

2. No

22) Do you collaborate with the department identified in Question 16 in the treatment and management of your hepatitis C positive patients?

- 1. Yes
  2. No **(skip to question 25)**

23) How would you rate your collaboration with the department identified in
Question 16 in the treatment and management of your hepatitis C positive patients?

**1 2 3 4 5**

**Minimal Some Active**

**Collaboration Collaboration Collaboration**

24) Please, briefly describe the ways in which you collaborate with the department identified in Question 16 in the treatment and management of hepatitis C patients.

________________________________________________________________________________________________________________________________________________________________________________________________________________________

**ADDITIONAL RESOURCES:**

25) Have you visited the VA’s National Hepatitis C Program’s website (www.hepatitis.va.gov)?

1. Yes
2. No

26) If “Yes,” did you find this website helpful? (Check ALL that apply)

- Yes
- No
- I downloaded products/materials from the website.
- I found what I was looking for easily.
- I had difficulty finding what I was looking for on the website.

**Thank you for completing this form. Please fax it to: Nancy Rettmann at 612-467-5312. *Your participation in the Liver Health Training Program will be confirmed upon receipt of this needs assessment questionnaire.***
